# Theft and Reception of Host Cell's Sialic Acid: Dynamics of *Trypanosoma Cruzi Trans*-sialidases and Mucin-Like Molecules on Chagas' Disease Immunomodulation

**DOI:** 10.3389/fimmu.2019.00164

**Published:** 2019-02-06

**Authors:** Leonardo Marques da Fonseca, Kelli Monteiro da Costa, Victoria de Sousa Chaves, Célio Geraldo Freire-de-Lima, Alexandre Morrot, Lucia Mendonça-Previato, Jose Osvaldo Previato, Leonardo Freire-de-Lima

**Affiliations:** ^1^Laboratório de Glicobiologia, Instituto de Biofísica Carlos Chagas Filho, Universidade Federal do Rio de Janeiro, Rio de Janeiro, Brazil; ^2^Laboratório de Imunomodulação, Instituto de Biofísica Carlos Chagas Filho, Universidade Federal do Rio de Janeiro, Rio de Janeiro, Brazil; ^3^Laboratório de Pesquisa em Tuberculose, Faculdade de Medicina, Universidade Federal do Rio de Janeiro, Rio de Janeiro, Brazil; ^4^Laboratório de Imunoparasitologia, Fundação Oswaldo Cruz, Rio de Janeiro, Brazil

**Keywords:** *Trypanosoma cruzi*, *trans*-sialidase, mucin-like molecule, sialic acid, glycan-binding protein, infectious disease, T-cell response

## Abstract

The last decades have produced a plethora of evidence on the role of glycans, from cell adhesion to signaling pathways. Much of that information pertains to their role on the immune system and their importance on the surface of many human pathogens. A clear example of this is the flagellated protozoan *Trypanosoma cruzi*, which displays on its surface a great variety of glycoconjugates, including *O*-glycosylated mucin-like glycoproteins, as well as multiple glycan-binding proteins belonging to the *trans*-sialidase (TS) family. Among the latter, different and concurrently expressed molecules may present or not TS activity, and are accordingly known as active (aTS) and inactive (iTS) members. Over the last thirty years, it has been well described that *T. cruzi* is unable to synthesize sialic acid (SIA) on its own, making use of aTS to steal the host's SIA. Although iTS did not show enzymatic activity, it retains a substrate specificity similar to aTS (α-2,3 SIA-containing glycotopes), displaying lectinic properties. It is accepted that aTS members act as virulence factors in mammals coursing the acute phase of the *T. cruzi* infection. However, recent findings have demonstrated that iTS may also play a pathogenic role during *T. cruzi* infection, since it modulates events related to adhesion and invasion of the parasite into the host cells. Since both aTS and iTS proteins share structural substrate specificity, it might be plausible to speculate that iTS proteins are able to assuage and/or attenuate biological phenomena depending on the catalytic activity displayed by aTS members. Since SIA-containing glycotopes modulate the host immune system, it should not come as any surprise that changes in the sialylation of parasite's mucin-like molecules, as well as host cell glycoconjugates might disrupt critical physiological events, such as the building of effective immune responses. This review aims to discuss the importance of mucin-like glycoproteins and both aTS and iTS for *T. cruzi* biology, as well as to present a snapshot of how disturbances in both parasite and host cell sialoglycophenotypes may facilitate the persistence of *T. cruzi* in the infected mammalian host.

## A Snapshot of the Nature of *Trypanosoma cruzi* Surface Coat

*Trypanosoma cruzi* presents a complex life cycle spanning two hosts, the hematophagous triatomine, and susceptible mammals (1). Throughout evolution, *T. cruzi* developed the capacity to adapt to hostile environments in both kinds of hosts. An important feature that was certainly decisive for the parasite adaptation to different hosts, as well as different niches within each host, was its ability to remodel its own surface coat (2, 3). It is well established that the cell surface of *T. cruzi* is composed by a wide variety of glycosylphosphatidylinositol (GPI)-anchored glycoconjugates expressed on a developmental stage-specific manner[(4–7).

Regarding the cell coat of the *T. cruzi* forms found in mammals, several studies revealed that it is mainly composed by both glycoinositolphospholipids (GIPLs) and heavily *O*-glycosylated mucin-like molecules (8, 9).

In addition, proteins belonging to *trans*-sialidase (TS) family (10–14); trypomastigote small surface antigen (TSSA) (15–17) and members of a multigenic family identified during the sequencing of the *T. cruzi* CL Brener genome, named mucin-associated surface proteins (MASPs) are found to a lesser extent (18–22).

## Sialic Acid-Containing Glycans Modulate the Establishment of *T. cruzi* Infection in Mammals' Cells

**Figure 1:**
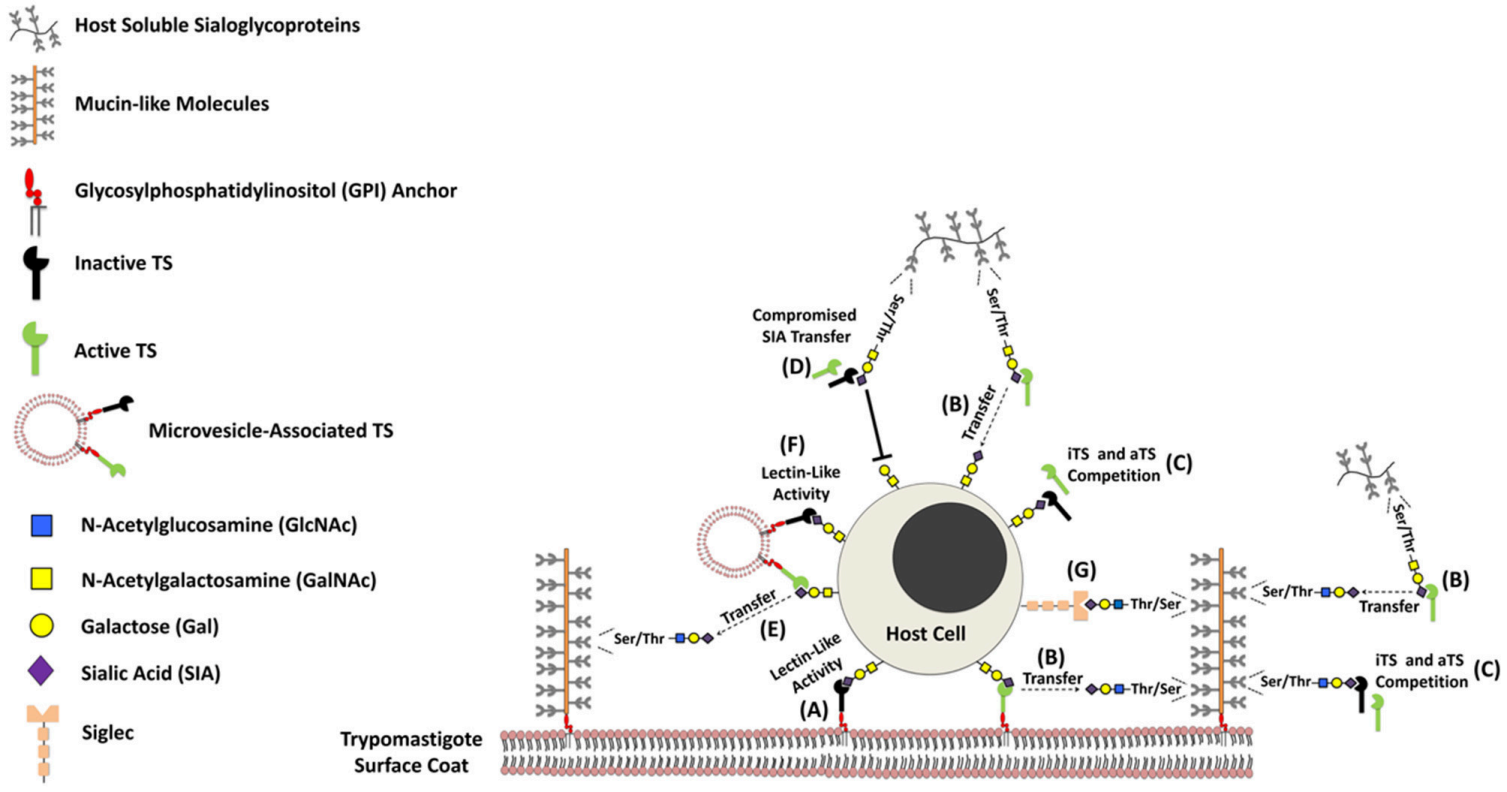
Schematic model showing the presence of *trans*-sialidases and mucin-like molecules on the parasite cell surface. The biological properties of both GPI-anchored proteins (*trans*-sialidases [TS] and mucin-like molecules) have been extensively studied over the last years, and their immunobiological functions have been gradually disclosed. *Trypanosoma cruzi* expresses on its surface both inactive (iTS) and active (aTS) TS proteins, that present similar substrate specificity (α-2,3 SIA). While iTS displays lectinic-like activity **(A)**, aTS shows the ability to modulate the sialoglycophenotype of both parasite and host cell glycans **(B)**. Since both TS proteins compete by α-2,3 sialo-containing glycans **(C)**, it may attenuate and or abrogate the process of SIA transfer mediated by aTS **(D)**. Consequently, it might be able to compromise biological phenomena depend on the catalytic activity displayed by enzymatically active members. In addition, both TS may be found associated to microvisicles, displaying the same properties mediated by both fully soluble enzyme **(E, F)**. The sialylation of glycoproteins found in the parasite cell surface besides to promote protection against soluble factors of the host immune system, may also provide ligand for SIA-binding proteins expressed by host cells, such as Siglecs **(G)**. Since this phenomenon compromises the effective function of immune cells, it may represent an interesting mechanism to guarantee the perpetuation of the parasite in their infected host.

Over the last twenty years, it has been known that simple, as well as complex carbohydrates (glycans) may play major structural, physical and metabolic roles in biological systems (23). Such functions include self/non-self-discrimination, ensuring correct protein folding, cell-to-cell signaling, cell adhesion and even differentiation, among others (24–27). The immune system, akin to the legions protecting the Roman Empire, is poised to defend the body against pathogens and transformed cells alike. One of the most important carbohydrates when it comes to the immune system is sialic acid (SIA) (28–30). More specifically the *N*-acetyl neuraminic acid (Neu5Ac). Immune responses deflagrated against *T. cruzi* are of particular interest, since the parasite is incapable of synthesizing SIA (31, 32). That would put *T. cruzi* squarely in the crosshairs of their mammal hosts' immune systems, since they somewhat rely on SIA to identify pathogens (3, 33, 34). The use of TS provides an elegant mechanism through which *T. cruzi* poaches SIA molecules from the hosts' cells and covers its own surface molecules, effectively creating a molecular ghillie suit to hide from mammalian phagocytes, posing a difficulty for the generation of an effective immune response (35–37). In addition to the enzymatically active members (aTS), which are able to modify the glycophenotype of both parasite and host cells (3, 13, 38, 39), TS also presents an inactive form (iTS), due to the naturally occurring Tyr342 → His substitution, which completely abolishes TS enzymatic activity (40). Despite the lack of catalytic function, it still plays an important role in *T. cruzi*-host cell interaction due to its lectinic activity (41–45) (Figure 1). Both extracellular (axenic) amastigote and trypomastigote forms of *T. cruzi* are infective to mammal cells (46–48). Regarding the trypomastigote forms, both iTS and aTS are GPI-anchored surface proteins (49). Recent findings revealed that sialylated mucins are present in lipid-raft-domains far away from TS molecules are found. By using unnatural sugar approach as chemical reporters, the authors demonstrated that the sialylation event is orchestrated by micro-vesicle-associated aTS instead of a membrane-anchored or fully soluble enzyme (34).

The importance of SIA-containing glycans on *T. cruzi*-host cell interplay was suggested over twenty-five years ago, when the authors demonstrated that the parasite's ability to penetrate into SIA-deficient cells was reduced when compared with wild-type cell lines (50). After this finding, many groups began investigating the events triggered by TS *in vitro* and in murine models (3, 37, 51–53).

## *trans*-Sialidases as Key Regulators of the Immune Evasion

Studies have shown that *T. cruzi* can recapitulate transient thymic aplasia in infected mice. It occurs in an early moment of the infection and aTS was proven responsible for the induction of apoptosis, since recombinant aTS alone can induce the alterations. In other studies, neutralizing anti-TS antibodies and the use of inhibitors prevented these effects (54). Also, an earlier study showed that recombinant iTS was incapable of eliciting these abnormalities (55). A study from Risso and colleagues demonstrated that the level of thymic damage was dependent on the parasite strain. More lethal strains (TcVI: RA, Q501, Cvd, and TcII: Br) present markedly higher levels of TS than their non-lethal counterparts (K-98, Ac and Hc - TcI) (56, 57). A different study showed that aTS does not appear to provoke thymocyte apoptosis directly. Instead, such effect seems to be centered on the thymic nurse cell complex, a region of the thymus cortex that contains mainly double-positive thymocytes, the most affected by TS (58). It is interesting to point out the studies that showed the pro-apoptotic effect was due to the alteration of the sialylation profile of target cells. By using lactitol, a competitive inhibitor that compromises the transfer of the sialyl residue to endogenous acceptors, but not the hydrolase activity of the enzyme, disallowed *ex vivo* and *in vivo* apoptosis caused by aTS (54). Years later, Lepletier and colleagues proposed that the apoptosis provoked by TS activity might also be capable of provoking an imbalance in the hypothalamus-pituitary-adrenal axis of *T. cruzi-*infected mice, leading to increased release of glucocorticoids, notorious immunossuppressants (59).

Early studies in the 90's already provided evidence of how aTS modulates the host immune system. Chuenkova and Pereira demonstrated that sensitizing mice with TS from conditioned supernatants, as well as recombinant aTS lead to higher parasitemia levels, and increased mortality rates. They also proposed that since animals with severe combined immunodeficiency, which lack functional T and B lymphocytes, were not affected. The logical conclusion was that TS was somehow affecting essential effector components of the adaptive immune system (60).

T lymphocytes must be activated to build up an effective response against invading organisms (61). This process involves loss of SIA residues in α-2,3 bonds from *O*-linked oligosaccharides, exposing free β-1,3 galactose (Gal) residues (62, 63). Such residues can be detected by the use of *Peanut agglutinin* lectin (PNA), which binds to terminal nonreducing Galβ1,3-GalNAc containing-sequences (64). That said *T. cruzi*'s flagship enzyme unique ability to transfer SIA residues springs to mind as the perfect candidate to interfere with this process. Our group demonstrated this by showing that in a TS-free infection, i.e., *Plasmodium berghei*-infected mice, activated CD8^+^ T cells exhibited a great number of terminal β-Gal residues, while in the presence of aTS, such residues were re-sialylated (37) (Figure 1). While further investigation is necessary, it is safe to say that such an effect would be a great help to the parasite, as dampening the cellular response, would help ensure the protozoa's survival within the host. Further evidence of that statement is found in the work of Pereira-Chioccola et al. (65). The authors describe how anti-alpha-Gal antibodies, purified from chronic Chagas disease patients, strongly bind to α-Gal terminals in mucins, causing severe structural perturbations that lead to parasite lysis, while sialylation by TS activity diminishes the damage. The authors proposed that the negative charge provided by SIA helps stabilizing the *T. cruzi* surface coat by electrostatic repulsion (65).

Although it has been known for more than twenty years that both iTS and aTS have almost identical structures and compete for the same substrate (40, 42, 44), little is known about the biological effects triggered by iTS during *T. cruzi* infection.

In an interesting report, Pascuale et al. (45) demonstrated that the expression of iTS gene in iTS-null parasites was able to improve *T. cruzi* invasion into Vero cells and increased their *in vivo* virulence as shown by histopathologic findings in skeletal muscle and heart tissue of *T. cruzi*-infected mice (45). Although the molecular mechanisms have not been elucidated, the authors claim that iTS might play a different or complementary pathogenic role to aTS (45). Recently, our group demonstrated that mice treated with an elevated (non-physiological) concentration of recombinant iTS showed a compromise of T cells homing to the cardiac tissue during *T. cruzi*-infection (44). Since iTS is capable of recognizing SIA-containing glycans, which are carried by many glycoproteins involved in leukocyte extravasation through activated venular walls (66–68) it would be plausible to speculate that iTS, through its lectinic property, may bind to sialylated peripheral homing receptors, impairing the homing of inflammatory cells to the target tissues. The poor development of genetic tools to directly dissect the biological roles displayed by either iTS or aTS, leads researchers towards alternative approaches for this technical deadlock. The use of both recombinant *T. cruzi-*iTS and aTS, separately or together, may provide a good way for studying the effects triggered by both TS proteins (44). Over the last fifteen years, studies demonstrated that when administered separately, both iTS and aTS elicit similar biological effects (42, 69, 70). However, until recently, there was no published data showing their combined effects. Immunological studies carried out by our group revealed that in *T. cruzi*-infected mice, the intravenous administration of high concentrations of recombinant aTS was able to modulate the expression of inflammatory signals by splenic T cells (44). Nevertheless, when both recombinant iTS and aTS were injected in equivalent amounts, such phenomena were significantly compromised (44). Additional studies are necessary to confirm our previous findings, however, it is plausible to speculate that when present in a soluble form and/or associated to microvesicles (34), iTS may compete with aTS by the same SIA-containing glycotopes and attenuate/abrogate biological events depending of the addition and/or removal of SIA residues.

Another question that needs addressing is the degree to which iTS is able to attenuate or abrogate biological events induced by aTS. In 2010, Freire-de-Lima and colleagues demonstrated that CD8^+^ T cells from *T. cruzi*-infected mice treated with a high concentration of recombinant iTS, became positive for PNA. These results reinforce the idea that iTS competes with aTS for SIA-containing glycotopes, then compromising an expected re-sialylation phenomenon that naturally happens during *T. cruzi* infection (37).

## *Trypanosoma cruzi* Mucins

*Trypanosoma cruzi* mucins are the parasite's most abundant surface glycoproteins. First described by Alves and Colli in epimastigotes, these highly glycosylated GPI-anchored mucin-like proteins were named A, B, and C glycoproteins (71). These proteins display a great deal of heterogeneity, with the genes responsible for encoding them being divided into two major families (3, 9, 72–74). The *T. cruzi* small mucin gene (TcSMUG) family encodes proteins that are expressed in the insect stages of the parasite's life, being essential to the infectivity on the insect host (75), while the TcMUC family, comprising from five to seven hundred genes, encodes the proteins expressed in the mammalian host. These proteins contain well-conserved N- and C-terminal regions, corresponding to ER and GPI anchor signals, respectively (72, 74, 76). This family can be further divided into three groups: (i) TcMUC I possesses a central domain with tandem repeats, with consensus sequences for *O*-glycosylation sites and it is more expressed in amastigotes (72, 73, 77); TcMUC II, found in trypomastigotes, displays a smaller number of repeats but is rich in serine and threonine residues (9, 72–74). Finally, TcMUCIII refers solely to the expression of a small surface protein, TSSA, or trypomastigote small surface antigen, being expressed only on cell-derived trypomastigotes (15). These mucin-like molecules contain a great number of *O*-linked oligosaccharides that are the main acceptors of SIA in the parasite's surface (Figure 1) (78–81). Unlike the classical vertebrate mucins, these oligosaccharides are linked to the protein core through α-GlcNAc residues, instead of α-GalNAc (82). Regardless, they contain a great number of free terminal β-Gal residues, which serve as ideal SIA acceptors (7, 78–81) (Figure 1). The *O*-linked oligosaccharides composition and size vary depending both the parasite strain (9, 78–80, 83–85) and its sialylation might promote immunosuppressive properties (please, see below).

The GPI-mucins expressed by *T. cruzi*, also known as sialoglycoproteins, are mucin-like molecules that are highly glycosylated and present a conserved GPI-anchor linked to the parasite cell surface (9, 80–87). All mucin GPI-anchors are constituted by a similar glycan core (Manα1-2Manα1-2Manα1-6Manα1-4GlcN) (9, 80, 85, 87). Except for the cell-derived trypomastigotes, where a branch of Gal residues can modify the GPI anchor (9, 84). The GPI-mucin lipid anchor differs according to the parasite's stage (80, 81, 85). In non-infective insect-derived epimastigotes, they are composed of saturated fatty acids; in metacyclic trypomastigotes, they are mainly inositol-phosphoceramides, and in the cell-derived trypomastigotes, they are composed wholly of alkylacyl-phosphatidylinositol (PI) structures, frequently insaturated (C18:1 or C18:2) (84, 85).

There is abundant data showing that following the early stages of *T. cruzi* infection, the patterns of resistance or susceptibility may be determined before adaptive immunity elements have a chance to respond, with components of the innate immune response playing crucial roles for parasite control (88). *T. cruzi* makes use of an expanded array of molecular strategies to invade an extensive range of host cells, as well as to avoid the host's immune defense. In the infection site, *T. cruzi* triggers the production of chemokines and pro-inflammatory cytokines, such as interleukin-12 (IL-12) and tumor necrosis factor-a (TNF-α), and the highly reactive oxygen and nitrogen species produced by cells of the Mϕ lineage (84, 85, 89–91). Over the last fifteen years, it has been described that GPI anchors expressed in the surface of *T. cruzi* are determinant in this process (85, 92, 93). In 2006, Bafica and colleagues demonstrated that the activation of innate immune response by *T. cruzi*-derived DNA and GPI anchors from trypomastigote mucins (tGPI-mucins anchors) forms, was able to promote the production of proinflammatory signals (84, 94). The authors revealed that the parasite's DNA stimulates cytokine production by Mϕ in a Toll-Like Receptor-9 (TLR9) dependent mechanism, and synergizes with parasite-derived tGPI-mucins, a TLR2 agonist, in the induction of IL-12 and TNF-α (94). More recently, it has been demonstrated that both living *T. cruzi* trypomastigote forms, as well as tGPI-mucins are able to induce high levels of IL-12 by human monocytes. Additionally, it has been proven that such effect depends on CD40-CD40L interaction and IFN-γ (95). In that work the authors claim that the polarized T1-type cytokine profile observed in *T. cruzi-*infected individuals might be a long-term effect of IL-12 production induced by lifelong exposure to *T. cruzi* tGPI-mucins (95).

It is well accepted that a great array of GPI-mucin genes is responsible for the variability of parasite cell surface (2). In 2004, an interesting work carried out by Buscaglia and collaborators demonstrated that the vast majority of the tGPI-mucin molecules found on the surface of the cell-derived trypomastigotes belong to the TcMUC II group. In this study, for the first time, the authors presented high evidence that multiple products of TcMUC II are concurrently expressed, suggesting that such molecules might represent a sophisticated strategy for the parasite to dampen the host immune response (9).

In 2002, Argibay and co-authors transfected higher eukaryotic cells (Vero cells) with TCMuc-e2 gene, which encodes for a mucin that is expressed in the blood-circulating stage of the parasite. The authors demonstrated that when transfected cells were exposed to human lymphocytes, an event of T cell anergy was observed. In this study, it was also demonstrated that the effect could be reversed by the addition of exogenous IL-2 (35). A different study discussed the effect of the interaction between the *T. cruzi* AgC10, a mucin-like molecule expressed by metacyclic trypomastigotes, as well as on amastigotes (96) and L-selectin in T cell surface. In an event independent of IFN-γ and nitric oxide, it was capable of inhibiting T cell proliferation and IL-2 secretion, as well as impairing IL-2 mRNA expression in response to mitogens. In fact, most genes whose expression is controlled by NFAT (Nuclear Factor of Activated T-cells) were affected and the overexpression of NFAT refuted the effects mediated by the parasite's glycoprotein (97).

The carbohydrate chains of mucin molecules are usually long extended structures (98). Over the last ten years has been demonstrated that the *O*-linked oligosaccharides composition of *T. cruzi* mucin-like molecules might exert direct effect on the host immune system. Since epimastigote forms are easier to be cultured *in vitro*, most of the studies investigating the biological roles triggered by *T. cruzi O*-linked glycans have been performed with non-infective forms for mammal cells. In 2013, Nunes and colleagues showed that a purified preparation of sialylated *T. cruzi* glycoproteins is capable of inhibiting clonal expansion as well as cytokine production by CD4^+^ lymphocytes. This happens through cell cycle arrest in the G1 phase and cannot be reversed by administration of exogenous IL-2, effectively rendering the cells anergic when stimulated through the T cell receptor (TCR) (99). The authors suggested that the starting point of this effect would be the interaction between the sialylated parasite mucins and Siglecs expressed on the T cell surface (Figure 1). An earlier study might substantiate this claim. Erdhmann and co-workers showed that the highly virulent *T. cruzi* Tulahuén strain was able to modulate the functionality of dendritic cells, through the interaction of its sialylated mucins with Siglec-E. The authors also confirmed that the desialylation of the parasite's surface molecules prevents such event (100).

## Possible Therapeutic Targets

The mucin-like proteins present in the surface of *T. cruzi* bear a distinct characteristic when compared to mucins or any other *O*-glycosylated protein on the surface of human proteins: the presence of galactofuranose (Gal*f*) residues (79). The flavoenzyme UDP-galactopyranose mutase (UMG) is not found in humans, but is essential to the composition of bacterial and fungal cell walls, as well as an important virulence factor for protozoa (6, 101, 102). A study in the late 80's even managed to show that anti-galactofuranose antibodies lead to a 70% inhibition of cell invasion (103). It should not come as a surprise that some groups treat UMG as an ideal therapeutic target, since the enzyme is not present in humans, and are working towards the development of UMG inhibitors (104–106). One study shows promise in halting the growth of some *Mycobacterium* species (107). It is important to note that this strategy suffers from a fundamental problem in the fact that so far Galf residues have not been found in the mucins expressed in the mammalian host stages'. The presence of Galf residues in metacyclics has been demonstrated (81).

*trans*-Sialidases also comes off as a potential drug target for the treatment or prevention of Chagas disease, and as such, many groups have been pursuing different strategies focused on TS as a target for either therapeutic or prophylactic methods. Good examples of this are recombinant proteins and DNA vaccines (108–111). Despite early reports showing that immunization with TS inhibits Th1 immune response (70), it was recently demonstrated that such a response can be elicited by the clever use of adjuvants (112). The same group has also shown that using the same model, aTS elicits stronger humoral and cellular responses than other *T. cruzi* antigens (113). Over the last decade, works from many research groups have demonstrated that vaccines candidates based on TS proteins are capable of protecting *T. cruzi*-infected mice (111, 114–118). Groundbreaking studies carried out by Rodrigues and Tarleton groups (119–122) have demonstrated that immunodominant CD8^+^ T cell immune responses directed to epitopes expressed by members of the TS family contribute to control *T. cruzi* infection, suggesting that non-antibody mediated cellular immune responses to the antigens expressed in the mammalian forms of *T. cruzi* might be used for the purpose of vaccination. In 2015, Pereira and collaborators started the development of both prophylactic and therapeutic vaccine protocols. The vaccines take advantage of the immunostimulation provided by a replication-defective human Type 5 recombinant adenoviruses (rAd) vector carrying sequences of amastigote surface protein-2 (rAdASP2), and TS (rAdTS). This strategy, rather offers a rational approach for re-programming the host immunity, achieving a more protective profile, leading to interruption of damage and even tissue recovery, particularly when it comes to chronic Chagas heart disease (123).

Another important focus field concerning *T. cruzi* TS is the search for effective inhibitors. A di-sialylated *N*-lactoside compound was shown to promote a 70% inhibition of TS activity through a competition mechanism (124). Sulfasalazine, a first line sulfa drug for rheumatoid arthritis, is also a moderate TS inhibitor. Although it does not lead to a great inhibition of the enzyme activity and it is not particularly toxic to the parasite strains tested by Lara-Ramirez's group, it is a good starting point for the development of new drugs, especially because sulfasalazine has been in use since the early 50s (125).

Several other researches have reported results on promising drugs, from competitive to non-competitive inhibitors, acting through reversible or irreversible mechanisms, some of those reaching up to 50% inhibition in the millimolar range (126–130).

An earlier work from our group has shown that 2-difluoromethyl-4-nitrophenyl-3,5-dideoxy-D-glycero-α-D-galacto-2-nonulopyranosid acid (NeuNAcFNP) is able to irreversibly inhibit TS in a time and dose-dependant manner. More importantly, it is able to produce a 90% inhibition of the infection of LLC-MK2 cells by *T. cruzi* Y strain trypomastigotes (131). Although it provides a unique form of inhibition and a chance for less major adverse effects, especially since TS bears no semblance with any human enzyme (132).

## Conclusion

In this review, we focused on the role of *T. cruzi* glycoconjugates and associated proteins in mediating the relationship between parasite and the human immune system. Throughout the years, several discoveries illustrated how TS, Tc-mucins and SIA are fundamental for the parasite to not only survive, but also thrive in an inhospitable environment like the human body. Mounds of evidence sustain the idea that TS is an important virulence factor, especially during the acute phase of the disease and is pivotal in aiding the parasite in bypassing the immune system. Authors also agree on the fact that mucins are major players in the balance between immune response and parasite survival, especially since it is the primary SIA acceptor in the protozoan membrane.

It is our belief that a better understanding of how *T. cruzi* is able to sabotage the human immune response will provide us with more effective tools to prevent and combat infections. Moreover, the parasite's unique system of handling SIA is almost certainly pivotal, since it involves a one-of-a-kind enzyme and an equally unique group of mucin-like proteins.

## Author Contributions

LF, KdC, VC, CF-d-L, AM, LM-P, JP, and LF-d-L participated in the writing of the paper.

### Conflict of Interest Statement

The authors declare that the research was conducted in the absence of any commercial or financial relationships that could be construed as a potential conflict of interest.
